# Comparison of WIFi, University of Texas and Wagner Classification Systems as Major Amputation Predictors for Admitted Diabetic Foot Patients: A Prospective Cohort Study

**DOI:** 10.5704/MOJ.2011.018

**Published:** 2020-11

**Authors:** PN Vera-Cruz, PP Palmes, LJM Tonogan, AH Troncillo

**Affiliations:** 1 Department of Internal Medicine, West Visayas State University Medical Center, Iloilo City, Philippines; 2 Department of Orthopaedics, West Visayas State University Medical Center, Iloilo City, Philippines

**Keywords:** diabetic foot, WIFi, wagner, University of Texas classification, major amputation

## Abstract

**Introduction:**

Classifications systems are powerful tools that could reduce the length of hospital stay and economic burden. The Would, Ischemia, and Foot Infection (WIFi) classification system was created as a comprehensive system for predicting major amputation but is yet to be compared with other systems. Thus, the objective of this study is to compare the predictive abilities for major lower limb amputation of WIFi, Wagner and the University of Texas Classification Systems among diabetic foot patients admitted in a tertiary hospital through a prospective cohort design.

**Materials and Methods:**

Sixty-three diabetic foot patients admitted from June 15, 2019 to February 15, 2020. Methods included one-on-one interview for clinico-demographic data, physical examination to determine the classification. Patients were followed-up and outcomes were determined. Pearson Chi-square or Fisher’s Exact determined association between clinico-demographic data, the classifications, and outcomes. The receiver operating characteristic (ROC) curve determined predictive abilities of classification systems and paired analysis compared the curves. Area Under the Receiver Operating Characteristic Curve (AUC) values used to compare the prediction accuracy. Analysis was set at 95% CI.

**Results:**

Results showed hypertension, duration of diabetes, and ambulation status were significantly associated with major amputation. WIFi showed the highest AUC of 0.899 (p = 0.000). However, paired analysis showed AUC differences between WIFi, Wagner, and University of Texas classifications by grade were not significantly different from each other.

**Conclusion:**

The WIFi, Wagner, and University of Texas classification systems are good predictors of major amputation with WIFi as the most predictive.

## Introduction

Type 2 diabetes is a rapidly growing epidemic. According to the World Health Organisation (WHO), its prevalence has nearly doubled from 4.7% to 8.5% of the global population between 1980 to 2014, affecting about 422 million people and most of the new cases are in low- and middle- income countries including the Philippines^1^. Diabetic foot is the most common lower extremity complication of diabetes and it occurs in at least 15% of diabetics in their lifetime preceding more than 80% of non-traumatic lower limb amputations^1^. The incidence is slightly higher in the Philippines where diabetic foot occurred in 19% of Type 2 diabetics shown in a study by Alcantara *et al*^2^. Diabetic foot and its sequalae account for billions of dollars in direct medical expenditures, as well as lengthy hospital stays and periods of disability^3,4^.

The risk for major amputation is multifaceted and is primarily determined by the degree of tissue loss, ischemia, and severity of foot infection as agreed upon by the American Diabetes Association and the Society for Vascular Surgery^5-7^. Classification systems are important tools in managing patients with diabetic foot. They aid in clinical decision-making, setting meaningful goals and expectations for patients and their families, decreasing length of hospital stay and economic burden of the disease by indicating prompt surgical intervention and avoiding unnecessary procedures^8,9^.

However, existing classification systems fail to adequately categorise the extent of tissue loss or the presence and severity of infection, thus none of the classification systems have been deemed as adequate to stratify patients with risk for lower limb amputation^8^. The Wagner classification system, although is well-established, does not account for severity of ischemia nor does it delineate gangrene due to infection versus ischemia^8^. Another commonly used classification proposed by University of Texas includes ischemia and infection but lacks severity gradation for each category^8,10^. Therefore, the Society for Vascular Surgery created WIFi (Wound, Ischemia, Foot infection) classification as a first step towards a comprehensive prognosticating tool for patients with risk for lower limb amputation. It proposed a scoring system that functioned much like the Tumour, Node, Metastasis staging of cancer and predicted risk for amputation. Prospective studies on WIFi are likewise scarce^5,11-20^.

Since the formulation of the WIFi classification system, there have been several validation studies conducted but these studies were mostly among Caucasian participants^13-21^. In West Visayas State University Medical Center, diabetic foot patients are classified according to Wagner Grade. However, a 2001 study by Oyibo *et al* showed that between Wagner Grade and University of Texas classification, the latter was more predictive of outcome^10^. Contrary to this, according to the study by Jeon *et al* in 2017, which evaluated five classification systems being used for diabetic foot, the Wagner grading was most predictive of lower limb amputation based on the Area Under the Receiver Operating Characteristic Curve (AUC) values used to compare the prediction accuracy versus the other four classification systems, including the one from University of Texas^20^.

Thus, this study was conducted with the hypothesis that the WIFi classification would have a comparable predictive ability as the Wagner classification and University of Texas classifications for major amputation in patients with diabetic foot.

## Materials and Methods

**Fig. 1:**
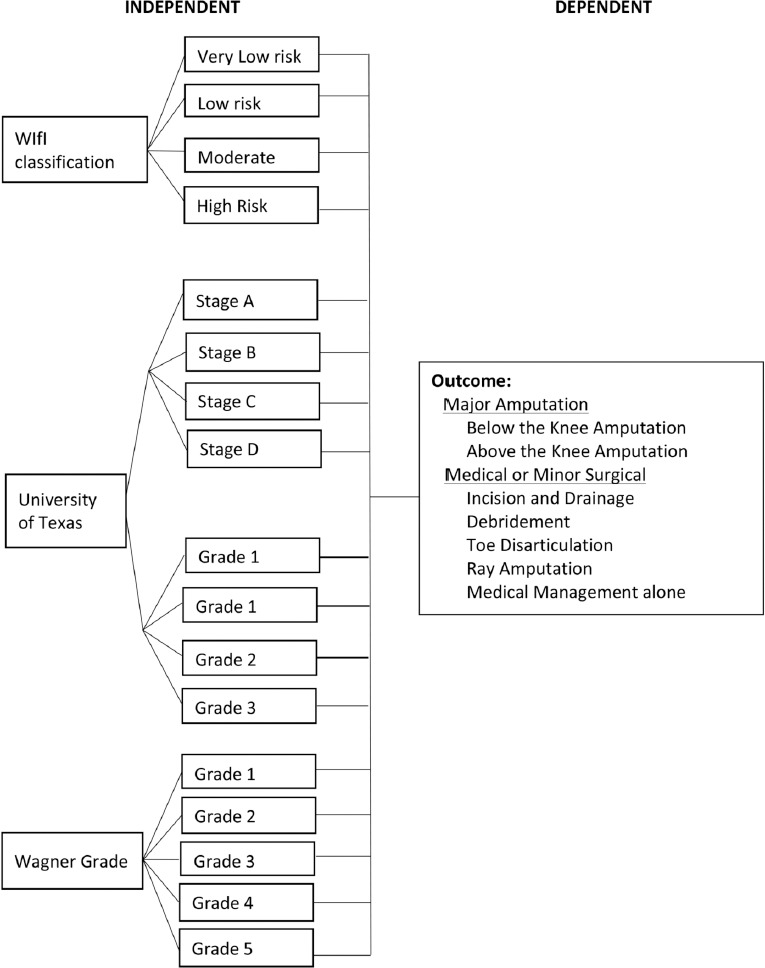
Conceptual Framework. Relationship Between Independent and Dependent Variables.

This is a prospective cohort study composed of 63 patients admitted at West Visayas State University Medical Center, Jaro, Iloilo City, Western Visayas, Philippines from June 2019 to February, 2019 who consented to participate and qualified based on the inclusion and exclusion criteria. The conceptual framework between the dependent and independent variables (Fig. 1). The one-year census of diabetic foot patients was 75 in 2018. We then used the following formula to calculate the sample size:


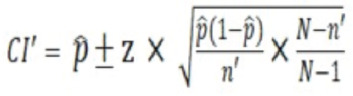


The protocol was submitted for ethics review and was approved. Notification and approval from the attending physician(s) of patients with Diabetic Foot were secured prior to inviting patients to join the study. The patients were invited if they were 19-year-old or more newly admitted for diabetic foot. Once approval was obtained, participants were given the informed consent form. Questions raised by the participants were answered. The participants were excluded if a) the treatment was not completed b) treatment was refused c) the participant was a trauma patient d) if the participant had an underlying malignancy, autoimmune disease, or pregnancy.

Methods for this study were patterned from the study by Oyibo *et al*, 2001 and the study by Jeon *et al,* 201710,20. The participants’ affected lower limbs were examined by the researchers to obtain the objective physical examination findings to simultaneously determine the WIFi classification, University of Texas stage, and Wagner grade based on the standard parameters of each system.

The WIFi classification has three components: Wound, Ischemia, and Foot Infection. Wound was graded as 1 if there was a small shallow ulcer on the distal leg or foot with no exposed bone, unless limited to the distal phalanx and no gangrene, 2 if there was a deeper ulcer with exposed bone, joint or tendon or gangrenous changes limited to the digits, 3 if there was extensive, deep ulcer involving forefoot and/or midfoot; deep, full thickness heel ulcer ± calcaneal involvement or with extensive gangrene involving the forefoot and/or midfoot; full necrosis ± calcaneal involvement^5,11^. Ischemia was graded using ABI, a grade of 0 was given if ≥.80, 1 if 0.6-0.79, 2 if 0.4-0.59, and 3 is ≤0.39. Foot infection was graded 1 if at least 2 of the following signs of infection were present: local swelling or induration, erythema >0.5 to ≤2cm around the ulcer, local tenderness or pain, local warmth, or purulent discharge; a grade of 2 if there was involvement of deeper structures deeper than skin and subcutaneous tissues but no systemic inflammatory response signs; and a score of 3 if there were signs of Systemic Inflammatory Response Syndrome manifested by two or more of the following: Temperature >38° or <36°C, heart rate >90 beats/min, temperature >38° or <36°C, heart rate >90 beats/min, respiratory rate >20 breaths/min or PaCO2 <32mm Hg, or white blood cell count >12,000 or <4000 cu/mm or 10% immature (band) forms. The grades of each variable were then plotted on the standard table for predicting risk for amputation to determine the WIFI classification^5^.

The University of Texas classification system (also assesses ulcer depth, the presence of wound infection, and the presence of clinical signs of lower-extremity ischemia) uses a matrix of grade on the horizontal axis and stage on the vertical axis. The grades of the University of Texas system are as follows: grade 0 (pre-or post-ulcerative site that has healed), grade 1 (superficial wound not involving tendon, capsule, or bone), grade 2 (wound penetrating to tendon or capsule), and grade 3 (wound penetrating bone or joint). Within each wound grade there are four stages: clean wounds (stage A), non-ischemic infected wounds (stage B), ischemic non-infected wounds (stage C), and ischemic infected wounds (stage D)^11^.

The Wagner classification has six grades, grade 0 for no open lesions, grade 1 for total destruction of the thickness of the skin, grade 2 if ulcer penetrated through skin, fat, and ligaments, but not affect bone, grade 3 if there was involvement of deeper tissues with abscess, osteomyelitis, or tendonitis, grade 4 if there was limited necrosis in toes or the forefoot necrosis of the complete foot, grade 5 if there was necrosis of the whole foot^11^.

**Fig. 2:**
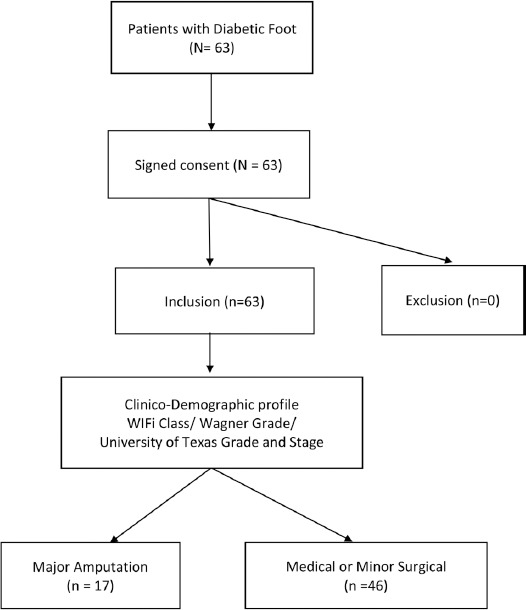
Flowchart for the Conduct of the study.

Two researchers – one internist and one orthopaedic surgeon resident physicians– scored the patients. Fig. 2 shows the flowchart for the conduct of the study. To address inter-observer variability, researchers performed the physical assessment on each participant and recorded their data on individual data collection sheets. The percent agreement and the kappa coefficient with 95% confidence interval was then calculated for each component to determine interobserver agreement with results showing an almost perfect agreement score of 0.892. Aside from this one to one patient-physician encounter to gather data for the clinico-demographic profile and determining the classification, daily rounds are carried out from admission to discharge. No intervention to the management was done.

Outcomes were measured as: a) Medical or minor surgical: incision and drainage plus antibiotics, debridement, digital removal, toe disarticulation, ray amputation, medical management alone; b) Major amputation: below the knee amputation and above the knee amputation.

Frequency and percentage to express categorical data and compared with the Chi-square test, Fisher’s exact test, or Cramer’s V, as appropriate. Pearson Chi Square determined the significant association between the WIFi, Wagner, and University of Texas classifications with the management outcomes of patients with diabetic Foot. Multivariate analysis determined any potential confounding factors and univariate ANOVA was used to determine their effect on the study outcomes. All statistical computations were computed with the use of statistical packages for social science (SPSS) software set at <0.05 level of significance.

## Results

Sixty-three patients were invited and enrolled in the study. As seen in Table I, of the 17 underwent major amputation, while 46 underwent management other than major amputation either medical management only, minor surgical procedures, or minor amputations. The age of the participants ranged from 41 to 80 years old with a mean age of 60 + 9.39SD. Forty (63.49%) participants were male and 23 (36.51%) were female. Forty-nine (77.78%) were ≤10 packyear smokers at the time of interview. Fifty-two (82.53%) were diagnosed with diabetes mellitus type 2 ≤ 10 years before developing diabetic foot ulcer. Fifty-six (88.89%) of the participants had no history of coronary artery disease and 34 (53.97%) had no renal insufficiency at the time of assessment. There was no statistically significant difference in the distribution of demographic characteristics and comorbidities among the participants except for duration of hypertension (p=0.001), duration since being diagnosed with diabetes (p=0.009), and ambulatory status (p=0.006) as seen in Table I. No dropouts occurred during the conduct of the study.

**Table I:** Clinico-demographic profile of participants (N=63)

Variables	Medical or minor surgical n= 46 (%)	Major amputation n=17 (%)	p-value
**Age (years)**			
41-60	25 (54.35)	10 (58.82)	0.216
61-80	21 (45.65)	6 (35.29)	
>80	0	1 (5.88)	
**Sex**			
Male	31 (67.39)	9 (52.94)	0.290
Female	15 (32.61)	8 (47.06)	
**Duration since diabetes mellitus diagnosis (years)**			
≤10	33 (71.74)	7 (41.18)	0.009
11-20	9 (19.57)	3 (17.65)	
>20	4 (8.70)	7 (41.18)	
**Tobacco use (in packyears)**			
non-smokers	18 (39.13)	8 (47.06)	0.985
≤10	16 (34.78)	5 (29.41)_	
11-20	5 (10.87)	2 (11.76)	
>20	7 (15.22)	2 (11.76)	
**Duration of hypertension (years)**			
Non-hypertensive	18 (39.13)	4 (23.53)	0.001
≤10	24 (52.17)	3 (17.65)	
11-20	3 (6.52)	6 (35.29)	
>20	1 (2.17)	4 (23.53)	
**History of coronary artery disease**			
Yes	4 (8.70)	3 (17.65)	0.473
No	42 (91.30)	14 (82.35)	
**Renal insufficiency**			
Yes	21 (45.65)	8 (47.06)	0.921
No	25 (54.35)	9 (52.94)	
**Ambulation**			
Able to walk but with assistance	4 (4.00)	7 (41.18)	0.006
Able to walk without assistance	42 (91.30)	10 (58.82)	

As shown in Table II, of the three classification systems, the WIFi and Wagner classificatons showed significant association with major amputation (p value of 0.000). Since the University of Texas classification is a matrix with grade on the horizontal axis and stage on the vertical axis, ranking of the groups according to severity is problematic. Hence, this study followed the methods of Jeon *et al* where the criteria of the university of Texas was divided into two groups a) by grade, which groups participants according to wound depth, and b) by stage, according to infection and ischemia^20^. The University of Texas classification by stage did not show a significant association to outcome (p=0.070), but a significant association was found between University of Texas classification by grade and outcome (p=0.000).

**Table II:** Association of WIFi, University of Texas, and Wagner classifications with Outcomes

Classification system		Medical or minor surgical n=46 (%)	Major amputation n=17 (%)	p value
WIFi stratification	Very Low	7 (15.22)	0	0.000
	Low	10 (21.74)	0	
	Moderate	23 (50.00)	2 (11.76)	
	High	6 (13.04)	15 (88.24)	
University of Texas by stage	A	0	0	0.070
	B	33 (71.74)	7 (41.18)	
	C	1 (2.17)	1 (5.88)	
	D	12 (26.09)	9 (52.94)	
University of Texas by grade	0	0	0	0.001
	1	13 (28.26)	0 (17.65)	
	2	19 (41.30)	3 (82.35)	
	3	14 (30.43)	14 (82.35)	
Wagner grade	0	0	0	0.000
	1	6 (13.04	0	
	2	18 (39.13)	1 (5.88)	
	3	11 (23.91)	3 (17.65)	
	4	9 (19.57)	4 (23.53)	
	5	2 (4.32)	9 (52.94	

Table III shows the Area under the Receiver Operator Characteristics Curve (AUC) of the three classification systems. WIFi and Wagner classifications showed significant predictive ability for major amputation with an AUC above 0.800, wherein WIFi classification showed an AUC of 0.899, while for Wagner classification it was 0.852. University of Texas classification showed a significant AUC of 0.785 (p value = 0.000) when the limbs were analysed by grade, but when it was analysed by stage, it did not show a significant predictive capability with an AUC of 0.575 (p=0.411).

**Table III:** Predictive Capability of WIFi, Wagner and the University of Texas classifications for major amputation among patients with diabetic foot

Classification systems	Area under the receiver operating curve (AUC)	p value
WIFi	0.899	0.000
Wagner	0.852	0.000
University of Texas by grade	0.785	0.000
University of Texas by stage	0.575	0.411

**Fig. 3:**
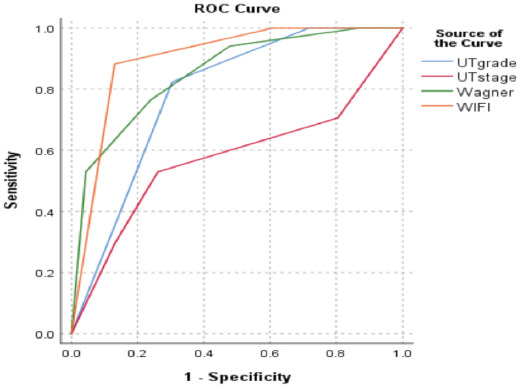
Receiver Operating Characteristic (ROC) curves of the three classification systems.

Fig. 3 shows the receiver operating characteristic (ROC) curves of the classification systems by plotting the true positive rate (sensitivity) against the false positive rate (1 – Specificity). WIFi ROC curve was closest to the ideal zone of highest sensitivity and specificity at the left upper quadrant of the graph. Wagner classification and University of Texas classification by staging show similar curves also approaching the ideal zone. Table IV shows the paired analysis of the AUCs of the classification systems. The University of Texas classification by stage showed a significantly different AUC compared to WIFi and Wagner classifications, and the University of Texas classification by grade. However, classifications by WIFi, Wagner, and the University of Texas by grade which showed significant predictive abilities for major amputation did not show significant difference in their AUCs despite WIFi having the highest AUC.

**Table IV:** Paired analysis of Area Under the Receiver Operating Characteristic Curve (AUC) of the classification systems

Classification systems	AUC difference	2-tail p value
WIFi – UTstage	0.324	0.000
Wagner - UTstage	0.277	0.015
UTstage - UTgrade	0.209	0.038
WIFi - UTgrade	0.114	0.052
Wagner - UTgrade	0.068	0.061
WIFi - Wagner	0.047	0.465

Selection bias was minimised by classifying each patient using the three systems. Information bias was also minimised by conducting a one-on-one interview with all of the patients for their clinicodemographic profile using an approved directed questionnaire; also, interrater variability was measured with a Kappa coefficient of 0.892. Multivariate analysis was done to identify possible confounders which showed that ambulation status has statistically significant main effect on the outcome (Hotelling’s Trace = 0.268, F (4,44) = 2.949, p = 0.030).

A separate ANOVA was conducted at an alpha level of 0.05 that examined the effect of ambulation status on the outcomes for each classification system which showed that ambulation status did not have a significant interaction on the outcomes of the study (pWIFi = 0.660, Wagner pwagner = 0.686, pUTstage = 0.526, pUTgrade = 0.364). Other clinic-demographic factors such as sex (p=0.765), duration of diabetes (p=0.192), duration of hypertension (p=0.370), history of coronary artery disease (p=0.075), renal insufficiency (0.0.120), and tobacco use (p= 0.176) did not have significant effect on the study outcomes.

## Discussion

In this study, the overall major amputation rate of diabetic foot patients was 26.98% (n=17). This is similar to other studies with overall amputation rates ranging from 26.2% to 30%^1^. This is expected in this study since most patients seek admission because the primary factors for foot amputation have already increased in severity i.e. larger wound area, infection, and ischemic changes. Furthermore, diabetic foot is the most common lower extremity complication of Type 2 diabetic patients. It occurs in at least 15% of diabetics in their lifetime and precedes more than 80% of non-traumatic lower limb amputations^3^.

Risk factors for major amputation have been extensively researched already and influences decision-making with regards to the need for major amputation^5,22-25^. In this study, three clinical characteristics produced a significant association with the management outcome: duration since diagnosed with hypertension (p value 0.001), duration since diagnosed with diabetes (p value 0.009) and ambulatory status (p=0.006).

Hypertension is a known promoter of peripheral vascular atherosclerosis and development of renal insufficiency, which are known risk factors for diabetic foot ulcers. According to the International Journal of Hypertension in 2013, hypertension and diabetes coexist in 40-60% of the time^26^. In a study by Quilici *et al*, hypertension was one of the most common comorbidities present among their diabetic foot patients with a rate of 72% second only to neuropathy with a rate of 91%^27^. Despite the prevalence, it did not show a significant association with major amputation in their study. The rate was lower in a study by Surriah *et al* involving 120 diabetic foot patients, hypertension also did not show a significant association with major amputation^28^. In the Thailand Diabetes Registry study the prevalence of hypertension among diabetics was 78.4% but it’s association with major amputation was not determined^29^. After thorough literature search, no study has included the duration of hypertension in association with amputation risk. In this study, 76.09% of the patients were hypertensive and this parameter showed a significant association to major amputation (p value = 0.001). This could be consequential to the increase in prevalence seen among the Asian population especially among Filipinos. In a study by Du *et al*, hypertension has increased (18%) in prevalence over a decade albeit not as dramatic as diabetes. Their study also showed that among the Asian subgroups, the prevalence of hypertension was highest among Filipinos^30^.

In this study, the duration since being diagnosed with diabetes mellitus showed a significant association with major amputation. Though majority (63.49%) of participants in this study were diagnosed with diabetes ≤10 years before developing diabetic foot ulcer, 43.47% of those who had been living with diabetes mellitus for >10 years underwent major amputation. The duration of diabetes is a known risk factor for amputation with significant duration ranging from 5 years to 15 years. In a study by Shatnawi *et al*, the risk factor associated with major amputation was duration of diabetes of >15 years^31^. Furthermore, in a study by Boulton *et al*, risk of ulcers and amputations increases two- to fourfold with both age and duration of diabetes^7^.

Ambulatory status also showed a significant association with major amputation. In this study, 60% of the patients who underwent major amputation were participants who could only walk with assistance. This may be due to inherent gait and soft tissue changes seen among diabetic patients that reduces the foot’s ability to accommodate for ambulatory ground reactive force to increase plantar pressure. According to the study by Wrobel *et al* entitled Diabetic Foot Biomechanics and Gait Dysfunction, diabetic patients exhibit a conservative gait where patients have a slower walking speed, wider base of gait, and prolonged double support time that promote repetitive trauma due to walking activities which exposes the foot to moderate or high pressure and shear forces^32^. This, coupled with neuropathy, soft tissue changes such as glycosylation of foot connective tissue, thinner fat pads, and stiffer joints put diabetic patients at risk of diabetic foot ulceration^33^.

Age and male gender were associated with 1.6-fold increase in foot ulcer risk in a study by Boulton *et al*^7^. These associations, however, were not seen in this study despite most of the participants were elderly >60 years old (53.85%) and most were male (61.54%) (page = 0.422, psex = 0.425, (Table I). This could reflect cultural and socioeconomic differences between the populations studied since previous studies were mostly done for Caucasian patients. In this study, the duration of diabetes mellitus showed a significant association with major amputation. A possible reason for this is that majority of participants in this study were just recently diagnosed with diabetes where 16 (61.54%) diagnosed ≤10 years before developing diabetic foot ulcer because in a study by Shatnawi *et al*^31^ the risk factor associated with major amputation was duration of diabetes of >15 years. Poor glycaemic control as shown by an elevated HbA1c >8.0% is also found to be associated with major amputation. However, this determination was not routinely done for the participants in the study, thus it was not included. Moreover, in a 2018 study by Kim *et al*, although improved glycaemic control reduced length of hospital stay, it did not reduce major lower extremity amputation making this marker a less reliable variable for risk for amputation^9^.

Cigarette smoking is also known to increase the risk of diabetes and peripheral arterial disease, and it has been associated with delays surgical healing in both elective and emergent diabetic foot surgery. A smoking history of >20 years was one key factor that was shown to lead to a more proximal amputation in 40% of cases in one study^21^. Results of a systematic review and meta-analysis by Liu *et al*, 2018, suggested that smoking increased the risk of diabetic foot amputation^22^. In this study however, most of the participants had an insignificant smoking history with most having a history of ≤20 packyears (65.39%, p = 0.675).

In a study by Nishijima *et al*^24^, coronary artery disease [defined as documented (prior) myocardial infarction or coronary artery revascularisation, or patients with ongoing or history of medication for coronary artery disease] was present in 83 patients, including 82% of patients who underwent major amputation and 63% of non-major amputation. The prevalence was significantly higher in the major amputation group (p=0.042)^25^. However, in this study, majority (92.31%) of our patients also did not have any history of coronary artery disease thus a significant association was not seen.

Renal insufficiency is associated with an increased incidence of peripheral artery disease and for lower extremity amputation. A study by Margolis *et al* showed that patients from primary care clinics with renal insufficiency (defined as eGFR <60ml/min per 1.73 m^2^) have increased risk for diabetic foot ulcer or lower extremity amputation (p <0.001) after follow-up of around 2.4 years^23^. However, our study did not show significant association (p = 0.683) between renal insufficiency and major amputation. This would suggest that renal function may not be an independent factor for major amputation in diabetic patients.

The area under the ROC Curve was used to compare the prediction accuracy of the classification systems. In a study by Jeon *et al* which compared five classification systems and predictive factors for amputation, Wagner classification was the most predictive of lower extremity amputation with an AUC of 0.892. This was followed by the University of Texas classification by Depth/Grade with an AUC of 0.85620. In this study, WIFi and Wagner classifications, and University of Texas classification by Stage (by depth of wound) showed good predictive ability wherein the WIFi classification produced the highest AUC of 0.899 followed by the Wagner classification with an AUC of 0.852, and by University of Texas classification by grade with and AUC of 0.785. The findings in this study are similar with the study by Carro *et al* where they showed that WIFi correlated with University of Texas and St. Elian classification systems for major amputation. In paired analysis, the AUCs between the classifications showed that their predictive ability was not significantly different from each other^34^. Our findings are consistent with other validation studies showing that each of the classification systems are capable of predicting major amputation^20,24^.

The University of Texas classification also classifies diabetic foot according to wound depth, infection and ischemia and is said to be better predictor of major amputation than Wagner classification^11^. Difference in AUC between University of Texas grading and staging highlights the impact of infection as an independent risk factor for amputation. Infection is associated with poor outcomes^35,36^. In this study, infection was present in 96.92% of the participants.

The results of this study provide valuable data to support WIFi classification as a good predictor of major amputation and it is comparable to other predictive systems. However, more prospective studies involving larger populations are needed to improve its applicability as a tool for precise decision-making for physicians managing diabetic foot patients. Therefore, we would like to recommend an extension of the study so that a more definitive conclusion could be derived with regards to the predictive ability of the WIFi classification. With a longer follow-up study, additional variables can also be added like wound healing time for non-amputated patients. Since this study is only limited to admitted patients, a post-management WIFi classification can also be obtained from non-amputated patients to evaluate the ability to predict one-year major amputation risk and to check for the status of amputated patients.

## Conclusion

This study showed that the Wound, Ischemia, and Foot Infection (WIFi), Wagner, and University of Texas classification systems are good predictors of major amputation with WIFi as the most predictive classification system among the three.
